# Functional health, environmental engagement and mental wellbeing as interrelated components of healthy ageing: a cross-sectional comparative study of community-dwelling and institutionalized older adults

**DOI:** 10.1186/s12877-026-08063-9

**Published:** 2026-07-31

**Authors:** Péter Fritz, Laura Csány, Regina Krasznai, Zoltán Szatmári, Réka Fritz

**Affiliations:** 1 Institute of Lifestyle and Health Sciences, Faculty of Economics, Health Sciences and Social Studies, Károli Gáspár University of the Reformed Church in Hungary, Budapest, Hungary; 2 Department of Oto-Rhino-Laryngology and Head-Neck Surgery, University of Szeged, Szeged, Hungary

**Keywords:** Healthy ageing, Environmental engagement, Nature exposure, Mental wellbeing, Functional Movement Screen, Handgrip strength, Older adults, Institutionalized older adults, Cluster analysis, Network analysis

## Abstract

**Background:**

Healthy ageing is commonly defined in relation to functional ability, intrinsic capacity and environmental context. However, less is known about how environmental engagement, mental wellbeing, physical functional indicators and religious/spiritual resources combine into healthy ageing profiles across residential settings. This study examined these associations in community-dwelling and institutionalized older adults.

**Methods:**

This cross-sectional, exploratory comparative study included 136 older adults in Hungary: 78 community-dwelling participants and 58 residents of long-term care institutions. Environmental engagement was operationalized as self-reported weekly frequency of spending at least 30 min in natural environments. Mental wellbeing was assessed using the World Health Organization Five Well-Being Index (WHO-5), religiosity using the Centrality of Religiosity Scale (CRS), movement quality using the Functional Movement Screen (FMS), and muscular strength using handgrip dynamometry. Analyses included group comparisons, correlations, multiple regression, exploratory cluster analysis and an exploratory thresholded correlation network.

**Results:**

Community-dwelling participants were younger and showed more frequent nature exposure, higher wellbeing and better FMS scores than institutionalized participants, whereas institutionalized participants reported higher religiosity. Nature exposure was associated with WHO-5 wellbeing (*r* = 0.220, *p* = 0.010) and FMS (*r* = 0.320, *p* < 0.001). FMS was associated with handgrip strength (*r* = 0.320, *p* < 0.001). The wellbeing regression model explained only a modest proportion of variance (R² = 0.086), and the association between nature exposure and wellbeing did not remain statistically significant after adjustment (β = 0.162, *p* = 0.072). A three-cluster solution was retained as the most interpretable exploratory phenotype structure, despite k = 2 showing somewhat stronger numerical cluster-validity indices. In the exploratory network, FMS had the highest centrality, while religiosity was peripheral.

**Conclusions:**

The findings suggest that healthy ageing in this sample was characterized by multidimensional, heterogeneous and interrelated profiles rather than residential status alone. Functional movement quality and environmental engagement were associated with several healthy ageing indicators, but causal mechanisms cannot be inferred. The results should be interpreted as exploratory and hypothesis-generating, warranting confirmation in larger longitudinal and intervention studies.

**Supplementary Information:**

The online version contains supplementary material available at https://doi.org/10.1186/s12877-026-08063-9.

## Background

Population ageing is a major global public health challenge. The World Health Organization (WHO) defines healthy ageing as the process of developing and maintaining the functional ability that enables wellbeing in older age [1, 2]. In this framework, functional ability reflects the interaction between intrinsic capacity and the environments in which people live [3, 4]. This perspective moves beyond chronological age and disease counts, emphasizing the combined roles of physical, psychological, social and environmental resources in shaping wellbeing and independence in later life [3–5].

The present study uses the term functional health in a narrower operational sense than the full WHO construct of functional ability. Specifically, functional health was operationalized through two physical functional indicators: movement quality, assessed by the Functional Movement Screen (FMS), and muscular function, assessed by handgrip strength. These indicators relate to intrinsic capacity and physical functioning, but they do not capture the full WHO concept of functional ability, which also includes environmental fit, participation and the person-environment interaction. This distinction is important for interpreting the findings.

Environmental engagement is a key component of healthy ageing because older adults’ opportunities for activity, social participation and restoration are shaped by their physical surroundings [1, 3]. Contact with natural environments has been associated with multiple health-related outcomes, including psychological wellbeing, physical activity, perceived restoration and mental health [6–10]. In older adults, systematic reviews and population-based studies suggest that green space and nature exposure are associated with subjective wellbeing and lower risk of depressive or anxiety-related symptoms, although effects vary according to accessibility, quality and individual circumstances [7–10]. These findings support examining nature exposure as a potentially relevant environmental correlate, while recognizing that observational associations do not establish causality.

Mental wellbeing is also central to ageing research. Subjective wellbeing in later life is associated with health, functioning, adaptation and quality of life [11]. Environmental engagement may be linked to mental wellbeing through several possible pathways, including physical activity, social contact and stress restoration, but these pathways are not directly tested in all observational designs [6, 7, 10, 12]. Therefore, studies that jointly examine environmental, psychological and functional indicators can help clarify how these components are associated in real-world older adult populations.

Functional health is frequently assessed through indicators of strength, mobility and movement capability. Handgrip strength is a widely used marker of muscular function and health risk in older adults, with evidence linking lower grip strength to disability, frailty, morbidity and mortality [13–16]. Movement quality may provide complementary information because it reflects the integrated performance of mobility, stability, balance and neuromuscular control. The FMS was originally developed as a standardized assessment of movement patterns [17, 18], and reliability studies and systematic reviews suggest acceptable rater reliability when administered by trained assessors [19, 20]. Evidence in older adults remains more limited, but available work supports its potential relevance to physical fitness and functional assessment in later life [21].

Religiosity and spirituality may provide meaning, coping resources and social support in older age [22–25]. However, these constructs are not necessarily central determinants of every physical or mental health outcome. They may operate as contextual or adaptive resources whose importance varies by life circumstances, health status and residential environment. This distinction is particularly relevant when religiosity is included alongside functional and environmental variables in multidimensional ageing models.

Healthy ageing research often relies on variable-centred analyses, yet older adults are heterogeneous and may show distinct combinations of environmental, psychological, functional and adaptive resources. Person-centred phenotyping and network-informed approaches can complement traditional group comparisons by describing profiles and associative structures among ageing-related variables [26–32]. Nevertheless, such analyses are sensitive to sample size, variable selection and analytic thresholds; in cross-sectional studies, they should be treated as exploratory and hypothesis-generating rather than confirmatory.

### Aim of the study

The aim of this cross-sectional exploratory study was to examine associations among environmental engagement, mental wellbeing, selected physical functional indicators and religiosity in community-dwelling and institutionalized older adults. The study addressed four research questions: (1) do community-dwelling and institutionalized older adults differ in environmental engagement, mental wellbeing, functional health indicators and religiosity; (2) are these variables associated with each other; (3) can interpretable multidimensional healthy ageing phenotypes be identified; and (4) which variables occupy central positions in an exploratory thresholded correlation network?

## Methods

### Study design and setting

This was a cross-sectional, comparative and exploratory study of older adults in Hungary. Participants were recruited from community-based senior organizations and long-term care institutions. The design was intended to compare residential settings while also examining multidimensional associations among environmental engagement, mental wellbeing, religiosity and physical functional indicators related to healthy ageing.

### Participants and eligibility screening

A total of 136 older adults participated in the study, including 78 community-dwelling individuals and 58 residents of long-term care institutions. The inclusion criterion was age 60 years or older. This threshold was selected to include adults in the early older-age transition and to capture variability in functional, environmental and psychosocial characteristics that may emerge before the more commonly used age threshold of 65 years. We acknowledge that this choice may limit direct comparability with studies using 65 years or older as the inclusion criterion.

Participants were eligible if they were able to understand study information, provide written informed consent, complete interviewer-administered questionnaires and safely participate in functional assessments. Cognitive and physical eligibility screening was conducted by trained members of the author team, who had the professional qualifications required for the physical and questionnaire-based assessments. Screening was based on structured interviewer-administered interaction, direct observation during recruitment and testing, and evaluation of whether participants could understand questions, follow instructions and safely perform the FMS and handgrip procedures. Participants with apparent severe cognitive impairment, inability to provide informed consent, acute medical conditions or physical limitations preventing safe completion of the assessments were not included. No formal diagnostic cognitive screening instrument, such as the Mini-Mental State Examination or Montreal Cognitive Assessment, was administered; this is acknowledged as a limitation.

### Ethical considerations

The study protocol was reviewed and approved by the Research Ethics Committee of the Faculty of Humanities and Social Sciences, Károli Gáspár University of the Reformed Church in Hungary (Approval No. BTK-PI/12059/2025). All participants received verbal and written information about the aims and procedures of the study and provided written informed consent prior to participation. The study was conducted in accordance with the Declaration of Helsinki and applicable data protection regulations. Data were stored using coded identifiers.

### Data collection procedure and data completeness

The data collection procedure was designed to minimize missing data and participant burden. Physical assessments and interviewer-administered questionnaires were completed in immediate succession using a station-based workflow. The assessment stations were placed directly next to one another to accommodate the age and mobility characteristics of the sample. Trained research assistants guided participants continuously from one station to the next. Data were entered electronically immediately after assessment using individual participant codes, which allowed the research team to verify completion of the required measurements during data collection.

All primary variables used in the group comparisons, regression models and cluster analysis had complete data for the full analytical sample (*N* = 136). One ancillary physical activity variable, sport/recreational participation days, had one missing value; therefore, analyses involving this variable used available cases (*n* = 135). No imputation was performed.

### Measures

#### Mental wellbeing

Mental wellbeing was assessed using the World Health Organization Five Well-Being Index (WHO-5) [33]. The WHO-5 consists of five positively worded items assessing subjective wellbeing over the previous two weeks. Items are rated on a six-point scale, and raw scores were transformed to a 0-100 scale, with higher scores indicating better wellbeing. The WHO-5 has extensive international psychometric support [33]. Hungarian older-adult-specific psychometric evidence was not available to the authors for all instruments used in this study; therefore, interpretation relied on established international evidence and this is noted as a limitation.

#### Environmental engagement and nature exposure

Environmental engagement was operationalized as self-reported weekly frequency of spending at least 30 min in a natural environment. Participants were asked how many days per week they spent at least 30 min in natural outdoor settings. The 30-minute criterion was selected as a pragmatic, behaviourally interpretable minimum exposure period suitable for interviewer-administered data collection among older adults. It should not be interpreted as a validated biological threshold or a dose-response cut-off. The variable was used as a frequency-based indicator of regular nature exposure and environmental engagement.

#### Religiosity

Religiosity was assessed using the Centrality of Religiosity Scale (CRS) [34]. The CRS assesses the subjective importance of religious beliefs and practices and includes intellectual, ideological, public practice, private practice and experiential dimensions. In the present study, religiosity was treated as an operational indicator of religious/spiritual resources, not as a comprehensive measure of spirituality in its broader existential meaning.

#### Functional health indicators

Functional movement quality was evaluated using the Functional Movement Screen (FMS) [17–20]. The FMS includes seven movement tasks designed to assess mobility, stability, balance, coordination and movement asymmetries. Total scores range from 0 to 21, with higher values indicating better movement quality. The FMS was interpreted as a composite indicator of functional movement quality rather than a direct measure of the full WHO functional ability construct.

Handgrip strength was measured using a calibrated hand dynamometer in accordance with standardized grip-strength testing principles [35]. Participants performed three maximal voluntary contractions, and the best value was retained for analysis. Handgrip strength was used as an indicator of muscular function and physical capacity.

#### Additional health-related and behavioural variables

Physical activity was assessed using items from the short International Physical Activity Questionnaire (IPAQ-SF) and additional indicators of weekly walking frequency and sport/recreational participation [36]. Perceived social support was assessed using the Duke Social Support Index (DSSI) [37]. Additional variables included body mass index (BMI), self-rated health, symptom burden and nature relatedness, the latter reflecting subjective connection with nature [38]. Where Hungarian-specific psychometric evidence could not be verified, this was acknowledged as a limitation.

### Statistical analysis

All inferential analyses were performed using IBM SPSS Statistics, Version 31.0 (IBM Corp., Armonk, NY, USA). Supplementary cluster-validation and exploratory network tables were assembled from the exported SPSS output and verified against the master statistical workbook. Descriptive statistics were calculated for all variables. Continuous variables are presented as means and standard deviations (SD), and categorical variables as frequencies and percentages.

Normality of continuous variables was evaluated using Shapiro-Wilk tests and visual inspection of distributions. Between-group comparisons were conducted using Welch independent-samples t-tests for continuous variables when group variances and distributions required robust comparison, and Fisher’s exact test or chi-square tests for categorical variables. Effect sizes are reported as Cohen’s d for continuous comparisons and are interpreted descriptively.

Potential outliers were screened using standardized z-scores and visual inspection of distributions. Extreme values were checked against possible data-entry or measurement errors. No observations were excluded on the basis of outlier screening because the reviewed values were retained as valid observations in the analytical dataset.

Associations among environmental engagement, mental wellbeing, religiosity, physical activity and functional health indicators were examined using Pearson correlations. Multiple linear regression models were used to examine variables associated with WHO-5 mental wellbeing, FMS score and handgrip strength. Because of the cross-sectional design, regression coefficients were interpreted as associations rather than evidence of causal prediction.

Exploratory cluster analysis was conducted using five core standardized variables: nature exposure days, WHO-5 mental wellbeing, FMS score, handgrip strength and religiosity. Hierarchical cluster analysis using Ward’s method was first used to explore structure, followed by K-means clustering. Alternative solutions from k = 2 to k = 6 were compared using inertia, silhouette coefficients and gap statistics. The decision to retain the final cluster solution is reported in the Results section to distinguish analytic procedures from findings. Age, sex and residential status were examined descriptively after cluster formation and were not used to define clusters.

An exploratory thresholded Pearson correlation network was constructed to describe the associative structure among nature exposure, WHO-5 wellbeing, FMS, handgrip strength, religiosity, walking days and sport/recreational participation. Edges were retained when |r| >= 0.20. This threshold was chosen a priori as a permissive exploratory criterion to retain weak-to-moderate associations potentially relevant in a small multidomain ageing sample. To improve transparency, the full correlation matrix, retained edges and centrality metrics are provided in the Supplementary Material. Degree, strength, betweenness and closeness were interpreted descriptively. The network should not be interpreted as a causal model.

A two-tailed *p* value < 0.05 was considered statistically significant. All cluster and network analyses are presented as exploratory and hypothesis-generating.

## Results

### Participant characteristics

The study included 136 older adults: 78 community-dwelling participants and 58 institutionalized participants. Participant characteristics and unadjusted residential comparisons are presented in Table 1. Institutionalized participants were significantly older than community-dwelling participants (81.10 ± 7.82 vs. 72.44 ± 6.17 years, *p* < 0.001). This age difference should be considered when interpreting unadjusted residential comparisons.

**Table 1:** Participant characteristics by residential status

Variable	Community-dwelling (*n* = 78)	Institutionalized (*n* = 58)	*p*-value	Effect size / note
Age, years	72.44 ± 6.17	81.10 ± 7.82	< 0.001	Cohen’s d = 1.25
Female sex, *n* (%)	68 (87.2)	48 (82.8)	0.476	Fisher exact test
Body mass index, kg/m²	26.18 ± 4.45	26.78 ± 5.41	0.492	Cohen’s d = 0.12
Nature exposure, days/week	5.79 ± 2.19	4.16 ± 2.93	< 0.001	Cohen’s d = 0.65
Nature relatedness total	23.95 ± 3.99	23.95 ± 3.56	0.999	Cohen’s d = 0.00
WHO-5 mental wellbeing score, 0-100	79.32 ± 10.74	75.28 ± 11.78	0.042	Cohen’s d = 0.36
Functional Movement Screen score	13.15 ± 4.00	8.90 ± 5.01	< 0.001	Cohen’s d = 0.95
Handgrip strength, kg	21.14 ± 5.59	18.97 ± 7.47	0.066	Cohen’s d = 0.34
Self-rated health	3.81 ± 0.84	2.91 ± 0.94	< 0.001	Cohen’s d = 1.01
Symptom burden	14.95 ± 4.98	17.91 ± 5.45	0.0015	Cohen’s d = 0.57
Religiosity score	1.82 ± 1.37	2.66 ± 1.46	< 0.001	Cohen’s d = 0.59
Weekly walking frequency, days/week	5.24 ± 2.25	4.86 ± 2.66	0.379	Cohen’s d = 0.16
Sport/recreational participation, days/week	3.48 ± 2.54	2.57 ± 2.82	0.055	Cohen’s d = 0.34

Values are mean ± SD unless otherwise indicated. *P*-values refer to Welch tests for continuous variables and Fisher’s exact test for sex. Cohen’s d values are reported as absolute magnitudes for readability

Community-dwelling participants reported more frequent nature exposure, higher WHO-5 wellbeing and higher FMS scores than institutionalized participants. Institutionalized participants reported higher religiosity and greater symptom burden. No statistically significant group differences were observed in BMI, nature relatedness, walking frequency or handgrip strength. Sport/recreational participation showed a non-significant trend favouring community-dwelling participants.

### Correlation analysis

Key correlations are shown in Table 2, and the complete correlation matrix for the exploratory network variables is provided in Supplementary Table S3. Nature exposure was positively associated with WHO-5 mental wellbeing (*r* = 0.220, *p* = 0.010) and FMS movement quality (*r* = 0.320, *p* < 0.001). Nature exposure was also associated with walking days (*r* = 0.428, *p* < 0.001). FMS was positively associated with handgrip strength (*r* = 0.320, *p* < 0.001), walking days (*r* = 0.207, *p* = 0.016) and sport/recreational participation (*r* = 0.294, *p* = 0.001, *n* = 135). The FMS-WHO-5 association was statistically significant but below the prespecified network-retention threshold (*r* = 0.188, *p* = 0.028). Religiosity showed weak or non-significant associations with the functional and wellbeing indicators.

**Table 2:** Key correlations among environmental engagement, wellbeing, function and activity

Association	*r*	*p*-value	*n*	Interpretation / note
Nature exposure - WHO-5 mental wellbeing	0.220	0.010	136	More frequent nature exposure was associated with higher wellbeing
Nature exposure - FMS movement quality	0.320	< 0.001	136	More frequent nature exposure was associated with better movement quality
Nature exposure - walking days	0.428	< 0.001	136	Nature exposure clustered with walking frequency
FMS movement quality - handgrip strength	0.320	< 0.001	136	Better movement quality was associated with greater muscular strength
FMS movement quality - walking days	0.207	0.016	136	Walking frequency was weakly associated with FMS
FMS movement quality - sport/recreational participation	0.294	0.001	135	Sport/recreational participation was associated with FMS
FMS movement quality - WHO-5 mental wellbeing	0.188	0.028	136	Significant bivariate association; below |r| >= 0.20 network threshold

Correlations are Pearson coefficients. The full correlation matrix for network variables appears in Supplementary Table S3

### Regression analysis

Regression results are summarized in Table 3. The model for WHO-5 mental wellbeing explained a modest proportion of variance (R² = 0.086; adjusted R² = 0.051; F = 2.46; *p* = 0.036). Nature exposure had the largest standardized coefficient in this model, but the adjusted association did not reach conventional statistical significance (β = 0.162, *p* = 0.072). Religiosity was not significantly associated with mental wellbeing after adjustment (β = 0.024, *p* = 0.781).

**Table 3:** Regression models for wellbeing, movement quality and handgrip strength

Outcome	Model statistics	Key variable	Standardized beta	*p*-value
Mental wellbeing	R² = 0.086; adjusted R² = 0.051; F = 2.46; *p* = 0.036	Nature exposure	0.162	0.072
Mental wellbeing	R² = 0.086; adjusted R² = 0.051; F = 2.46; *p* = 0.036	Religiosity	0.024	0.781
Functional Movement Screen	R² = 0.310; adjusted R² = 0.278; F = 9.67; *p* < 0.001	Age	-0.323	< 0.001
Functional Movement Screen	R² = 0.310; adjusted R² = 0.278; F = 9.67; *p* < 0.001	Living environment	-0.230	0.012
Functional Movement Screen	R² = 0.310; adjusted R² = 0.278; F = 9.67; *p* < 0.001	Nature exposure	0.147	0.066
Handgrip strength	R² = 0.539; adjusted R² = 0.517; F = 25.11; *p* < 0.001	Sex	-0.655	< 0.001
Handgrip strength	R² = 0.539; adjusted R² = 0.517; F = 25.11; *p* < 0.001	Age	-0.263	< 0.001
Handgrip strength	R² = 0.539; adjusted R² = 0.517; F = 25.11; *p* < 0.001	FMS score	0.168	0.021

Only key model terms are displayed. Coefficients are standardized beta coefficients. Models are interpreted as associative rather than causal

For FMS score, the model explained 31.0% of variance (R² = 0.310; adjusted R² = 0.278; F = 9.67; *p* < 0.001). Age was the strongest associated variable (β = -0.323, *p* < 0.001), and living environment also contributed significantly (β = -0.230, *p* = 0.012). Nature exposure showed a positive but non-significant adjusted association (β = 0.147, *p* = 0.066).

For handgrip strength, the model explained 53.9% of variance (R² = 0.539; adjusted R² = 0.517; F = 25.11; *p* < 0.001). Sex (β = -0.655, *p* < 0.001), age (β = -0.263, *p* < 0.001) and FMS score (β = 0.168, *p* = 0.021) were significant associated variables.

### Healthy ageing phenotypes

Cluster-number diagnostics are reported in Supplementary Table S2. The two-cluster solution showed the strongest numerical indices among the examined solutions (silhouette = 0.239; gap = 0.349), whereas the three-cluster solution had a lower silhouette (0.198) and gap statistic (0.276). The three-cluster solution was nevertheless retained because it provided a more differentiated and theoretically interpretable exploratory phenotype structure. The clusters should therefore be interpreted as exploratory healthy ageing profiles rather than validated diagnostic categories.

The retained three-cluster solution is summarized in Table 4. Cluster 1, labelled the Low-Engagement Institutional Adaptation Profile, included 38 participants (27.9%) and was characterized by lower nature exposure, lower mental wellbeing and less favourable functional indicators. Institutionalized participants predominated in this cluster. Cluster 2, labelled the Community Environmental-Functional Profile, included 56 participants (41.2%) and showed higher environmental engagement and favourable movement quality. Community-dwelling participants were overrepresented. Cluster 3, labelled the Integrated Resilient Healthy Ageing Profile, included 42 participants (30.9%) and combined high environmental engagement, higher wellbeing and favourable functional characteristics.

**Table 4:** Data-driven healthy ageing phenotypes from cluster analysis

Variable	Cluster 1: Low-Engagement Institutional Adaptation	Cluster 2: Community Environmental-Functional	Cluster 3: Integrated Resilient Healthy Ageing
*n *(%)	38 (27.9)	56 (41.2)	42 (30.9)
Nature exposure, days/week	2.97 ± 2.83	5.88 ± 2.15	5.98 ± 1.97
Mental wellbeing total	69.97 ± 13.00	79.23 ± 9.10	82.31 ± 8.89
Functional Movement Screen score	6.47 ± 4.61	13.02 ± 3.39	13.50 ± 3.82
Handgrip strength, kg	15.80 ± 4.73	19.64 ± 5.06	24.97 ± 6.63
Religiosity	2.92 ± 1.19	0.82 ± 0.77	3.31 ± 0.78
Age, years	82.58 ± 8.11	73.25 ± 6.39	74.14 ± 7.03
Female sex, %	100.0	89.3	66.7
Community-dwelling, %	18.4	80.4	61.9
Institutionalized, %	81.6	19.6	38.1

Values are mean ± SD unless otherwise indicated. Cluster labels are exploratory phenotype descriptors. Age, sex and residential status were examined descriptively and were not used in cluster formation

All three phenotypes included participants from both residential settings, indicating that residential status alone did not fully explain the multidimensional healthy ageing profiles.

### Exploratory correlation network

**Fig. 1:**
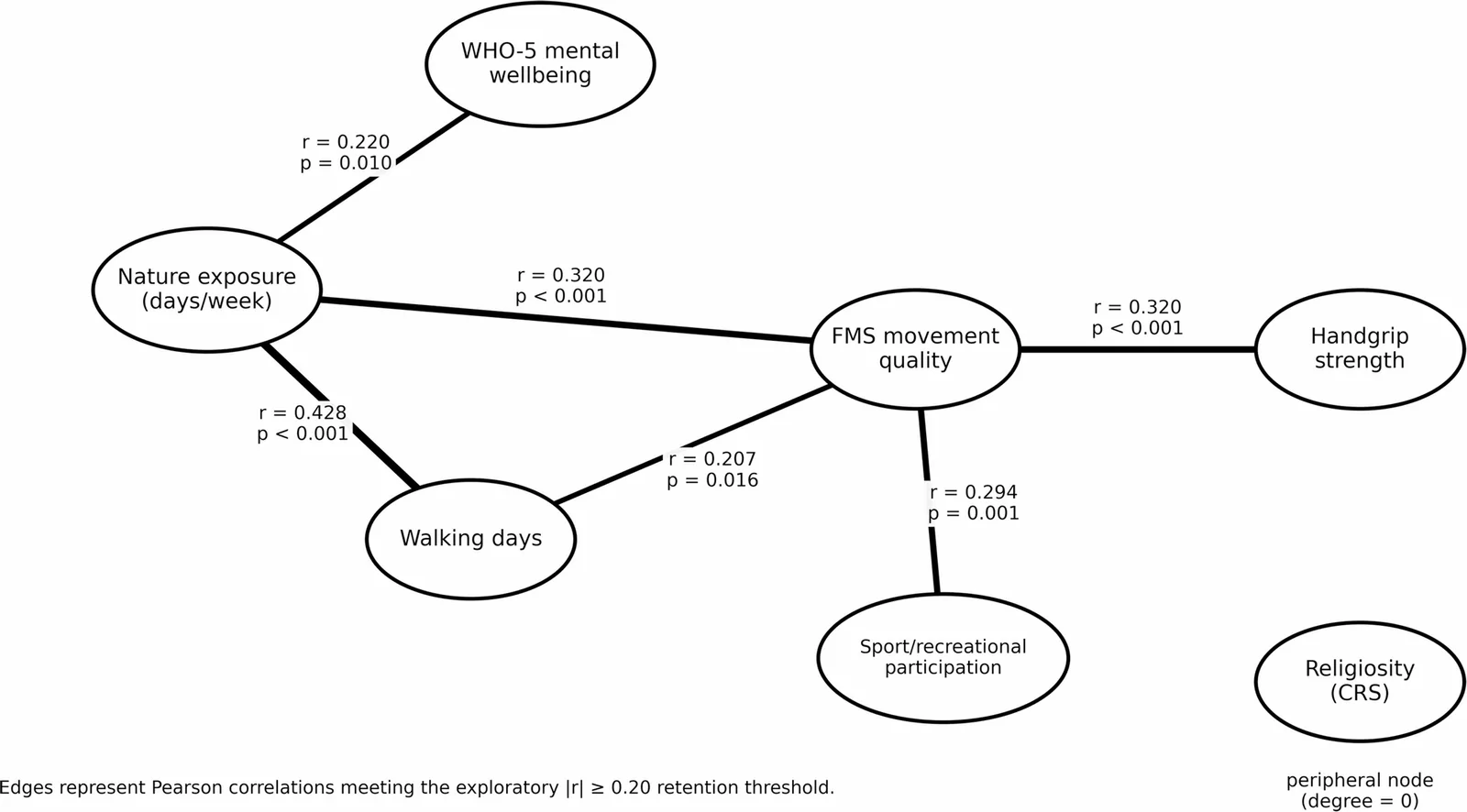
Exploratory thresholded correlation network. Revised exploratory thresholded Pearson correlation network. Solid edges represent correlations meeting the prespecified |r| >= 0.20 retention threshold. Edge labels show r values and *p* values. Religiosity is shown as a peripheral node because degree = 0 in the thresholded network. The figure is an exploratory association-based visualization and does not imply causal direction

The exploratory thresholded correlation network is shown in Fig. 1. Complete retained edges and centrality metrics are provided in Supplementary Tables S4 and S5. FMS had the highest centrality values (degree = 4, strength = 1.141, betweenness = 0.467, closeness = 0.694), indicating that movement quality occupied the most connected associative position in the thresholded network. Nature exposure had degree = 3, strength = 0.967, betweenness = 0.267 and closeness = 0.595, functioning as a secondary hub connected to wellbeing, FMS and walking frequency.

Walking days had degree = 2, while WHO-5 wellbeing, handgrip strength and sport/recreational participation each had degree = 1. Religiosity had degree = 0 in the thresholded network, suggesting that it functioned as a contextual or adaptive resource rather than a central node in the measured environmental-functional network. These findings are descriptive and exploratory and should not be interpreted as evidence of causal pathways.

## Discussion

### Principal findings

This study examined healthy ageing as a multidimensional, cross-sectional and exploratory construct in community-dwelling and institutionalized older adults. The findings suggest that environmental engagement, mental wellbeing and physical functional indicators were interrelated, while religiosity contributed to phenotype differentiation but was peripheral in the thresholded correlation network.

Several results require cautious interpretation. Community-dwelling participants showed more favourable unadjusted profiles for nature exposure, wellbeing and FMS, but they were also substantially younger than institutionalized participants. The bivariate association between nature exposure and wellbeing was significant, but the association weakened and did not reach statistical significance in the adjusted wellbeing regression model. The three-cluster solution was retained for theoretical interpretability despite the two-cluster solution showing somewhat stronger numerical cluster-validity indices. Finally, the network analysis was thresholded, descriptive and exploratory.

### Environmental engagement and mental wellbeing

The positive bivariate association between nature exposure and WHO-5 wellbeing is consistent with literature linking green space and nature contact to mental health and subjective wellbeing in older adults [6–12]. However, the adjusted regression model explained only 8.6% of the variance in wellbeing, and nature exposure did not remain statistically significant after adjustment. Therefore, the present findings should be interpreted as evidence of a modest bivariate association that was attenuated in multivariable analysis, not as evidence of an independent or causal effect.

Possible pathways linking nature exposure to wellbeing, such as physical activity, social interaction, stress restoration and perceived environmental quality, remain hypothesized mechanisms in the present study. They were not directly tested. Future longitudinal and intervention studies should examine whether and how structured access to natural environments is associated with changes in wellbeing among older adults in different residential settings.

### Functional health indicators

FMS movement quality was associated with nature exposure, handgrip strength, walking days and sport/recreational participation, and it had the highest centrality values in the exploratory network. These findings suggest that movement quality may be an informative integrative indicator of physical function within multidimensional healthy ageing research. However, this should not be equated with the full WHO concept of functional ability, which also includes environmental interaction and participation [1, 3].

Handgrip strength was strongly explained by sex and age and was also associated with FMS in the regression model. This aligns with evidence that grip strength is a clinically meaningful marker of muscular function and health risk in older adults [13–16, 35]. The present results suggest that movement quality and muscular function capture related but distinct aspects of functional health.

### Healthy ageing phenotypes

The phenotype analysis identified three interpretable profiles, but these profiles should be treated as exploratory. The two-cluster solution showed stronger numerical validity indices, while the three-cluster solution provided a more differentiated representation of environmental, psychological, functional and religious/adaptive characteristics. This trade-off is common in exploratory person-centred analyses, where interpretability and theoretical relevance must be weighed against numerical diagnostics [27–29].

The finding that all three phenotypes included participants from both residential settings supports the view that older adults should not be treated as homogeneous groups based only on place of residence. Residential status was relevant, but not sufficient to explain the observed multidimensional profiles.

### Religiosity as a contextual and adaptive resource

Religiosity showed a distinct pattern. It differed between residential groups and contributed to cluster differentiation, but it was not a central node in the exploratory network and was not independently associated with mental wellbeing in the regression model. This suggests that religiosity may function primarily as a contextual or adaptive resource rather than as a central component of the measured environmental-functional network.

This interpretation is compatible with literature suggesting that religious and spiritual resources may support coping, meaning and adaptation in later life, particularly under conditions of illness, loss or institutionalization [22–25]. The present study did not test these pathways directly; therefore, religiosity should not be interpreted as a causal determinant of healthy ageing outcomes in this sample.

### Practical implications

The findings have practical implications for hypothesis generation and future intervention design. If causal relationships are confirmed in longitudinal or experimental work, programmes that improve safe access to natural environments, walking opportunities, therapeutic gardens or outdoor recreational activities may be relevant for supporting wellbeing and function in older adults. In the present cross-sectional study, these should be regarded as plausible intervention hypotheses rather than demonstrated effects.

Similarly, routine assessment of movement quality may be useful for identifying functional needs, but the current results do not prove that improving FMS would cause improvements in wellbeing or environmental engagement. Future studies should test whether multidimensional programmes addressing environmental access, physical function and psychosocial resources lead to measurable improvements in healthy ageing outcomes.

### Strengths and limitations

The study has several strengths. It applied a multidimensional healthy ageing framework, included both community-dwelling and institutionalized older adults, combined self-report and physical functional measures, and used complementary variable-centred and person-centred analytical approaches. Data collection was organized to minimize missing data, and the revised analysis provides transparent supplementary information on missingness, cluster diagnostics, correlation matrices and network centrality.

Several limitations must also be acknowledged. First, the cross-sectional design precludes causal inference and temporal ordering. Second, the sample size was modest for cluster and network analyses, and these results should be interpreted as exploratory and hypothesis-generating. Third, institutionalized participants were substantially older than community-dwelling participants, and age differences may have influenced several residential comparisons. Fourth, cognitive and physical eligibility were screened by trained researchers through structured interaction and observation, but no formal MMSE or MoCA screening was administered; subtle cognitive impairment therefore cannot be fully excluded.

Fifth, environmental engagement was assessed using a pragmatic self-report measure based on weekly frequency of at least 30 min spent in natural environments. This operationalization was not a validated dose-response threshold and should be interpreted cautiously. Sixth, Hungarian older-adult-specific validation evidence was not available to the authors for all instruments, including some functional, social and environmental measures. Seventh, the network used a permissive |r| >= 0.20 threshold and descriptive centrality indices; results may be sensitive to threshold choice and should be confirmed using larger samples and stability analyses.

## Conclusions

In this cross-sectional exploratory study, healthy ageing among community-dwelling and institutionalized older adults was characterized by heterogeneous and interrelated profiles of environmental engagement, mental wellbeing, selected physical functional indicators and religiosity. Nature exposure was associated with wellbeing and movement quality at the bivariate level, but its adjusted association with wellbeing was not statistically significant. FMS movement quality occupied the most central position in the exploratory thresholded correlation network, while religiosity appeared to function as a contextual/adaptive resource rather than a central network node.

The findings should be interpreted as exploratory and hypothesis-generating. Larger longitudinal and intervention studies are needed to test causal pathways, validate phenotype structures and determine whether multidimensional programmes targeting environmental access, movement quality and psychosocial resources can improve healthy ageing outcomes.

## Supplementary Information

Supplementary Material 1.

## Data Availability

The datasets generated and/or analysed during the current study are not publicly available because they contain potentially sensitive information from older adult participants and residential care settings, and unrestricted public sharing was not covered by the consent and ethical conditions under which the data were collected. De-identified data supporting the conclusions of this article may be made available from the corresponding author upon reasonable request and subject to applicable ethical, legal and institutional restrictions.

## Abbreviations

ANOVA: analysis of variance
BMI: body mass index
CRS: Centrality of Religiosity Scale
DSSI: Duke Social Support Index
FMS: Functional Movement Screen
ICOPE: Integrated Care for Older People
IPAQ-SF: International Physical Activity Questionnaire - Short Form
IQR: interquartile range
SD: standard deviation
WHO: World Health Organization
WHO-5: World Health Organization Five Well-Being Index
