# Supplementary material for: Functional health, environmental engagement and mental wellbeing as interrelated components of healthy ageing: a cross-sectional comparative study of community-dwelling and institutionalized older adults
Source: BMC Geriatr. 2026 Jul 31;26:1359. doi: 10.1186/s12877-026-08063-9 (PMC13717991; doi:10.1186/s12877-026-08063-9)
Supplement: Supplementary file 1 [file 12877_2026_8063_MOESM1_ESM.docx]

**Supplementary Material**

Manuscript: Functional Health, Environmental Engagement and Mental Wellbeing as Interrelated Components of Healthy Ageing

This supplementary material provides additional information requested during peer review: missing values, cluster-number diagnostics, the complete correlation matrix for the exploratory network variables, retained network edges and centrality metrics.

# Supplementary Table S1. Missing values by variable

| **Variable** | **Missing n** | **Missing %** |
| --- | --- | --- |
| Age | 0 | 0.0 |
| Sex | 0 | 0.0 |
| Living environment | 0 | 0.0 |
| Walking days | 0 | 0.0 |
| Sport/recreational participation days | 1 | 0.7 |
| Nature exposure days | 0 | 0.0 |
| Religiosity | 0 | 0.0 |
| WHO-5 mental wellbeing | 0 | 0.0 |
| Nature relatedness | 0 | 0.0 |
| Symptoms total | 0 | 0.0 |
| Self-rated health | 0 | 0.0 |
| BMI | 0 | 0.0 |
| FMS score | 0 | 0.0 |
| Handgrip strength | 0 | 0.0 |

Note. Primary variables used in the main group comparisons, regression models and cluster analysis had complete data for N = 136. Sport/recreational participation days had one missing value; analyses involving this ancillary variable used available cases (n = 135). No imputation was performed.

# Supplementary Table S2. Cluster-number diagnostics

| **k** | **K-means inertia** | **Silhouette** | **Gap statistic** | **Gap SE** |
| --- | --- | --- | --- | --- |
| 2 | 528.56 | 0.239 | 0.349 | 0.022 |
| 3 | 443.51 | 0.198 | 0.276 | 0.058 |
| 4 | 385.00 | 0.208 | 0.243 | 0.036 |
| 5 | 353.78 | 0.184 | 0.236 | 0.061 |
| 6 | 327.05 | 0.174 | 0.236 | 0.025 |

Note. The two-cluster solution showed the strongest numerical indices among the examined solutions. The three-cluster solution was retained in the revised manuscript because it provided a more differentiated and theoretically interpretable exploratory phenotype structure. The clusters should not be interpreted as externally validated diagnostic categories.

# Supplementary Table S3. Complete Pearson correlation matrix for exploratory network variables

| **Variable** | **Nature exposure** | **WHO-5 wellbeing** | **FMS** | **Handgrip** | **Religiosity** | **Walking days** | **Sport days** |
| --- | --- | --- | --- | --- | --- | --- | --- |
| Nature exposure | 1.000 | 0.220 (0.010; n=136) | 0.320 (<0.001; n=136) | 0.055 (0.526; n=136) | -0.100 (0.246; n=136) | 0.428 (<0.001; n=136) | 0.193 (0.025; n=135) |
| WHO-5 wellbeing | 0.220 (0.010; n=136) | 1.000 | 0.188 (0.028; n=136) | 0.182 (0.034; n=136) | -0.031 (0.716; n=136) | 0.070 (0.418; n=136) | 0.098 (0.260; n=135) |
| FMS | 0.320 (<0.001; n=136) | 0.188 (0.028; n=136) | 1.000 | 0.320 (<0.001; n=136) | -0.068 (0.430; n=136) | 0.207 (0.016; n=136) | 0.294 (<0.001; n=135) |
| Handgrip | 0.055 (0.526; n=136) | 0.182 (0.034; n=136) | 0.320 (<0.001; n=136) | 1.000 | 0.031 (0.724; n=136) | -0.047 (0.587; n=136) | 0.057 (0.514; n=135) |
| Religiosity | -0.100 (0.246; n=136) | -0.031 (0.716; n=136) | -0.068 (0.430; n=136) | 0.031 (0.724; n=136) | 1.000 | 0.006 (0.941; n=136) | -0.069 (0.428; n=135) |
| Walking days | 0.428 (<0.001; n=136) | 0.070 (0.418; n=136) | 0.207 (0.016; n=136) | -0.047 (0.587; n=136) | 0.006 (0.941; n=136) | 1.000 | 0.167 (0.053; n=135) |
| Sport days | 0.193 (0.025; n=135) | 0.098 (0.260; n=135) | 0.294 (<0.001; n=135) | 0.057 (0.514; n=135) | -0.069 (0.428; n=135) | 0.167 (0.053; n=135) | 1.000 |

Note. Cells show r (p-value; n). The exploratory network retained edges with |r| >= 0.20. Values involving sport/recreational participation days used n = 135 due to one missing value; other values used n = 136.

# Supplementary Table S4. Retained network edges

| **Node 1** | **Node 2** | **r** | **p-value** | **n** |
| --- | --- | --- | --- | --- |
| Nature exposure days | WHO-5 mental wellbeing | 0.220 | 0.010 | 136 |
| Nature exposure days | FMS score | 0.320 | <0.001 | 136 |
| Nature exposure days | Walking days | 0.428 | <0.001 | 136 |
| FMS score | Handgrip strength | 0.320 | <0.001 | 136 |
| FMS score | Walking days | 0.207 | 0.016 | 136 |
| FMS score | Sport/recreational participation days | 0.294 | 0.001 | 135 |

Note. Edges were retained when |r| >= 0.20. The network is a descriptive, exploratory thresholded Pearson correlation network and does not imply causal direction.

# Supplementary Table S5. Network centrality metrics

| **Node** | **Degree** | **Strength (absolute r)** | **Betweenness** | **Closeness** | **Interpretation** |
| --- | --- | --- | --- | --- | --- |
| Nature exposure days | 3 | 0.967 | 0.267 | 0.595 | Secondary hub connecting environmental engagement with wellbeing and functional movement |
| WHO-5 mental wellbeing | 1 | 0.220 | 0.000 | 0.379 | Peripheral but theoretically important wellbeing outcome |
| FMS score | 4 | 1.141 | 0.467 | 0.694 | Primary central node integrating environmental engagement, activity and muscular function |
| Handgrip strength | 1 | 0.320 | 0.000 | 0.417 | Functional output connected mainly through FMS |
| Religiosity | 0 | 0.000 | 0.000 | 0.000 | Isolated in thresholded network; contextual/adaptive resource dimension |
| Walking days | 2 | 0.635 | 0.000 | 0.521 | Activity/environmental node linking nature exposure and FMS |
| Sport/recreational participation days | 1 | 0.294 | 0.000 | 0.417 | Activity node connected to FMS |

Note. Centrality metrics are descriptive and should be interpreted cautiously because the network is exploratory, thresholded and based on cross-sectional data.
